# Endoscopic Transluminal Stent Placement for Malignant Afferent Loop Obstruction

**DOI:** 10.3390/jcm11216357

**Published:** 2022-10-27

**Authors:** Chinatsu Yonekura, Takashi Sasaki, Takafumi Mie, Takeshi Okamoto, Tsuyoshi Takeda, Takaaki Furukawa, Yuto Yamada, Akiyoshi Kasuga, Masato Matsuyama, Masato Ozaka, Naoki Sasahira

**Affiliations:** Department of Hepato-Biliary-Pancreatic Medicine, Cancer Institute Hospital, Japanese Foundation, 3-8-31, Ariake, Koto-ku, Tokyo 135-8550, Japan

**Keywords:** afferent loop obstruction, balloon-assisted enteroscopy, self-expandable metal stent, transluminal, surgically altered anatomy

## Abstract

Background: Malignant afferent loop obstruction (ALO) is rare condition and is difficult to manage. Endoscopic transluminal treatment has become easier with the advent of balloon-assisted enteroscopes with a large working channels and self-expandable metal stent (SEMS) with a 9 Fr delivery system. Methods: From July 2016 to March 2022, 22 patients with symptomatic malignant ALO who underwent endoscopic transluminal treatment (Initial cohort), of which 18 patients received endoscopic transluminal SEMS placement (SEMS cohort), were retrospectively evaluated. We evaluated outcomes of endoscopic transluminal treatment and long-term outcomes of SEMS placement for malignant ALO. Results: In the Initial cohort, technical and clinical success rates were both 95.5%. The median procedural time was 28.0 min. One case of guidewire-induced micro-perforation occurred as an early complication (4.5%). In the SEMS cohort, and no early complication was observed. Recurrent obstruction occurred in two cases (11.1%) during the follow-up period (median: 102 days). One was managed by additional SEMS placement and the other was treated conservatively. Conclusions: High technical and clinical success was achieved by endoscopic transluminal treatment with short procedural time for malignant ALO. Endoscopic SEMS placement also appears to be safe and effective, and additional SEMS placement can be considered in cases of re-obstruction.

## 1. Introduction

Malignant afferent loop obstruction (ALO) is a condition in which the afferent loop is obstructed with malignant tumors. The incidence of malignant ALO after gastrectomy or pancreaticoduodenectomy has been reported to be 0.05–4.3% [1,2,3,4]. Recently, malignant ALO has increased due to the increase in surgery for gastric, biliary and pancreatic cancers and advances of chemotherapy [4]. Abdominal pain, fever and jaundice are the major symptoms associated with cholangitis, pancreatitis and intestinal dilatation due to the stasis of the afferent loop. Without sufficient intestinal decompression, the condition can give rise to intestinal perforation, sepsis, and ultimately, death.

There are several treatment methods of intestinal decompression for malignant ALO [5]. While surgical bypass was previously the standard of care, minimally invasive treatment is desirable because malignant ALO usually occurs at an advanced stage. Two percutaneous approaches are available: direct puncture of the dilated afferent loop and puncture of the dilated bile duct. There is a high risk of peritonitis due to intraperitoneal leakage of the accumulated intestinal fluid (bile and pancreatic juices) with direct puncture of the dilated afferent loop, while bile duct dilation is necessary to perform percutaneous transhepatic biliary drainage (PTBD) [6]. If bile duct pressure is elevated, there is also a risk of intraperitoneal bile leakage during the PTBD procedure. Endoscopic drainage has recently become the favored approach for malignant ALO. There are three possible endoscopic approaches: the transluminal approach, the endoscopic ultrasound (EUS)-guided approach via intrahepatic bile ducts, and the EUS-guided direct approach to the dilated afferent loop. Recently, EUS-guided anastomoses using self-expandable metal stent (SEMS) have been introduced for the management of malignant ALO. However, the EUS-guided approach is only possible in selected institutions, as it is associated with the risk of leakage of accumulated intestinal fluid when a one-step device is not available.

The endoscopic transluminal approach for malignant ALO has been performed for a long time. In its early days, an endoscopic naso-drainage (END) tube was placed for temporary decompression [7]. With the advent of SEMS, endoscopic transluminal SEMS placement became available for malignant ALO. In the 2000s, through-the-scope (TTS) SEMS placement was only possible for simple gastrointestinal reconstruction which allowed an endoscope with a large working channel to pass [8]. In the early 2010s, in the cases of complex gastro-intestinal reconstruction, it was necessary to insert a balloon-assisted enteroscope (BAE) with a small working channel, advance a guidewire beyond the stenosis, remove the BAE with the overtube remaining in place, and placing SEMS through the overtube [9]. In the late 2010s, endoscopic transluminal stent placement was facilitated by slim delivery systems and a BAE with a large working channel, allowing for TTS SEMS placement even in cases with complex gastrointestinal reconstruction. However, reports on treatment outcomes of endoscopic transluminal stent placement and long-term outcomes of SEMS placement for malignant ALO are still limited. We therefore conducted a retrospectively study to clarify these points and performed a brief review of the existing literature.

## 2. Materials and Methods

### 2.1. Patients

Consecutive patients with symptomatic malignant ALO who required gastrointestinal decompression at our institution between July 2016 and March 2022 were extracted from our prospectively maintained database. The diagnosis of malignant ALO was based on the findings of afferent loop obstruction and of dilation of blind loop on abdominal computed tomography (CT). The diagnosis of malignancy was judged based on the clinical course, endoscopic findings, and CT findings. Pathological diagnosis from the stenotic site was not essential. The Initial cohort included all naïve cases undergoing endoscopic transluminal treatment for malignant ALO. The SEMS cohort included those in the Initial cohort which underwent SEMS placement for malignant ALO. Exclusion criteria were: (i) stent or tube placement using methods other than the endoscopic transluminal approach, (ii) cases with simultaneous gastrointestinal obstruction at other sites, and (iii) cases of gastrointestinal perforation before the endoscopic procedure.

### 2.2. Devices

A single-balloon enteroscope (SIF-H290S; Olympus Medical, Tokyo, Japan), a distal attachment (D-201-10704; Olympus Medical) and an overtube (ST-SB1S; Olympus Medical) was used as a BAE in this study. This enteroscope has a 9.2 mm distal end diameter, a 152 cm working length and a 3.2 mm working channel. A 6 Fr or 7.2 Fr END tube (SilkyPass; Boston Scientific, Marlborough, MA, USA) was used for external drainage. A 7 Fr double-pigtail stent (Through & Pass; Gadelius Medical, Tokyo, Japan, Zimmon Biliary Stent Sets; Cook Medical, Bloomington, IN, USA) was used when a plastic stent was selected for internal drainage. Several types of uncovered SEMS with 9 Fr delivery systems were used depending on the time of study period: Niti-S Colonic stent (TaeWoong Medical, Gyeonggi-do, South Korea), Niti-S Pyloric/Duodenal stent (TaeWoong Medical), HANAROSTENT Naturfit DUO stent (Boston Scientific), JENTLLY NEO Duodenal stent (Japan Lifeline, Tokyo, Japan), and NEXENT duodenal stent (Next Biomedical, Incheon, South Korea). The diameter of Niti-S Colonic stemt was 18 mm, while all other SEMS had diamaters of 22 mm. The lengths of uncovered SEMS were 60, 80, 100, 120 mm.

### 2.3. Endoscopic Procedure

**Figure 1:**
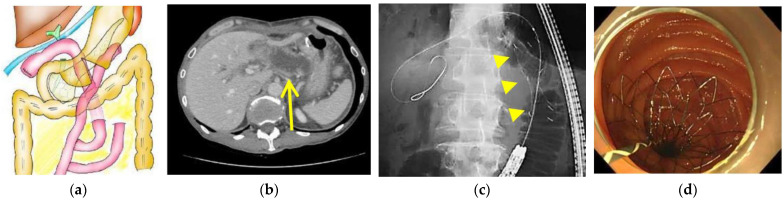
Endoscopic transluminal self-expandable metal stent placement for malignant afferent loop obstruction (Double bypass and type 1 obstruction case). (**a**) Illustration of bowel reconstruction, (**b**) Contrast-enhanced computed tomography showing dilation of the afferent loop (yellow arrow), (**c**) Fluoroscopic view showing a metal stent (yellow arrowheads) placed using the through-the-scope technique, (**d**) Endoscopic view after stent deployment.

All procedures were performed by expert endoscopists with over 10 years of experience in pancreatico-biliary interventional endoscopy or by trainees under their direct supervision. A BAE with an overtube was inserted into the afferent loop using CO_2_ insufflation under conscious sedation using midazolam and pethidine hydrochloride. The stenotic site was detected endoscopically and fluoroscopically. The BAE was advanced through the stenotic site if it could be passed without resistance. In such cases, the dilated afferent loop was quickly decompressed by suction, and locations of the papilla or the choledochojejunal anastomosis and/or the pancreaticojejunal anastomosis were identified endoscopically. When the BAE could not be advanced through the stenotic site, a catheter (MTW ERCP catheter; MTW Endoskopie Manufaktur, Wesel, Germany) and an 0.025-inch guidewire (Visiglide 2; Olympus Medical) were advanced through the stenotic site. In such cases, the approximate location of the papilla or the choledochojejunal anastomosis was identified fluoroscopically. In either case, the guidewire was inserted across the stenotic site, and the stricture length was evaluated fluoroscopically. Either an END tube, a plastic stent, or an uncovered SEMS was deployed along the guidewire using the TTS method under direct endoscopic vision and/or fluoroscopic imaging (Figure 1). When the afferent loop was severely dilated, a 6 Fr or 7.2 Fr END tube was temporarily placed, and an uncovered SEMS or a plastic stent was placed at a later date, after symptoms stabilized.

### 2.4. Data Collection

We retrospectively reviewed medical records of the enrolled patients. The following data was checked for the Initial cohort to evaluate the efficacy and safety of endoscopic transluminal approach: age, sex, Eastern Cooperative Oncology Group (ECOG) performance status (PS), primary disease, type of surgery, type of reconstruction, ascites, dilation of intrahepatic bile duct, obstruction type, ALO symptoms, time from surgery to onset of malignant ALO, use of antithrombotic agents and anti-vascular endothelial growth factor antibodies, and prior radiotherapy. The following procedural data was also collected: time to reaching the stenotic site, total procedure time, ability to advance the BAE that through the stricture, and drainage method. Technical and clinical success rates and early complications were also evaluated.

The following data was reviewed for the SEMS cohort to evaluate the long-term outcomes of SEMS: END before SEMS placement, stricture length, total procedure time, technical and clinical success rates, SEMS diameter and length, early and late complications, follow-up period, ALO recurrence, re-intervention, chemotherapy after stent placement, time to recurrent obstruction, and overall survival.

### 2.5. Definitions

Malignant ALO was classified into three types based on a previous report [8]: type 1, the obstruction site is located between bowel anastomoses of afferent loop (such as Y anastomosis site of Roux-en-Y reconstruction) to the papilla or the choledochojejunal anastomosis; type 2, the obstruction site involves the papilla or choledochojejunal anastomosis; type 3, the obstruction site is located between the choledochojejunal and pancreaticojejunal anastomoses.

Technical success was defined as the successful advancement of the BAE to the stenotic site and successful deployment of a decompression tube or stent across the stricture. Clinical success was defined as the improvement of the symptoms and the successful decompression of blind loop within 24 h. The time to reaching the stenotic site was calculated as the time from the BAE passing through the mouth to the time when the stenotic site was reached. Total procedure time was defined as the time from the BAE passing through the mouth to complete removal of the BAE [10,11,12].

Similar to a previous report on duodenal stenting, recurrent obstruction was defined as a composite endpoint of either SEMS occlusion or migration [13]. Time to recurrent obstruction was measured the day of SEMS placement to the date of diagnosis of recurrent obstruction. Patients who did not experience recurrent obstruction were censored at the date of the last follow-up or death. Overall survival was defined as the time from SEMS placement until death from any cause. Early and late complications was defined as any adverse events occurring within 7 days and more than 7 days after SEMS placement, respectively. The severity of adverse events was evaluated according to the American Society for Gastrointestinal Endoscopy’s grading system [14].

### 2.6. Statistical Analysis

Categorical variables are presented as absolute numbers and percentages. Continuous variables are presented as medians and ranges. A *p*-value < 0.05 was considered statistically significant. Time to recurrent obstruction and overall survival were calculated using the Kaplan-Meier method and compared with log-rank test, and presented as median and 95% confidence interval (CI). All statistical analyses were carried out using EZR ver. 1.40 (Saitama Medical Center, Jichi Medical University, Saitama, Japan) [15]. Follow-up data was confirmed up to 31 July 2022.

## 3. Results

### 3.1. Study Flow

**Figure 2:**
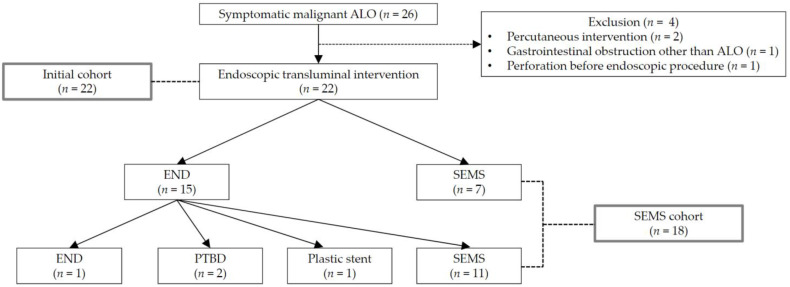
Study flow diagram. ALO, afferent loop obstruction; END, endoscopic naso-drainage; SEMS, self-expandable metal stent; PTBD, percutaneous transhepatic biliary drainage.

A study flow diagram is shown in Figure 2. A total of 26 patients were diagnosed with symptomatic malignant ALO and received first therapeutic intervention during the study period. Four patients were excluded, as detailed above. As a result, 22 patients were included in the Initial cohort. Of these, 18 patients received endoscopic transluminal SEMS placement and were also included in the SEMS cohort.

### 3.2. Initial Cohort

Characteristics of the 22 included patients are summarized in Table 1. The median age was 64.5 years (37–83 years). Thirteen patients (59.1%) were males, and 18 patients (81.8%) had ECOG PS of 0–1. Pancreatic cancer was the most common primary cancer (45.5%), and half of the patients (50.0%) received pancreaticoduodenectomy. All patient except one (95.5%) received open surgery. Dilation of intrahepatic duct was only observed in five patients (22.7%). The most common obstruction type was type 1 (68.2%). Abdominal pain (50.0%) and fever (40.9%) were the major symptoms of malignant ALO. The median period from surgery to ALO was 612 days (86–1806 days). Five patients (22.7%) were taking antithrombotic agents. Three patients on direct oral anticoagulants and one patient on warfarin discontinued these medications before the procedure. The one other patient remained on direct oral anticoagulants before and after the procedure. One patient received prior radiotherapy near the field of SEMS placement approximately two years prior. The patient was permitted to undergo SEMS placement because the stricture was located outside the irradiation field. One patient was treated with an anti-vascular endothelial growth factor antibody (ramucirumab) two years before SEMS placement.

Treatment outcomes of endoscopic transluminal intervention are summarized in Table 2. Median time required to reach the stenotic site was 10.5 min (range, 4–64 min). Median total procedure time was 28.0 min (range, 12–106 min). The BAE could be advanced through the stenotic site in 7 cases (31.8%). Seven patients (31.8%) underwent SEMS placement during the first endoscopic session. The remaining fifteen patients (68.2%) underwent END tube placement during the first session, of which 12 ultimately had END tubes replaced with plastic stents or SEMS in the second session and two underwent conversion to PTBD. One patient with total gastrectomy with Roux-en-Y reconstruction was converted to PTBD route due to type 2 obstruction. Technical and clinical success was not achieved in the other patient, in whom the tip of the END tube was inappropriately placed in the stomach because the type of reconstruction was unknown at the time of the procedure (later determined to be a double bypass). PTBD was therefore placed the next day, leading to successful decompression of the dilated afferent loop. The last END patient died of primary disease with the END tube in place. As a result, technical and clinical success rates were both 95.5% (21/22).

One case (4.5%) of guidewire-induced micro-perforation was the only early complication. This patient recovered by conservative therapy and a plastic stent was placed several days after was classified as a mild adverse event. No cases of stent migration were observed in this study.

### 3.3. SEMS Cohort

Eighteen patients were included in the SEMS cohort. Treatment outcomes of the first endoscopic transluminal SEMS placement are summarized in Table 3. Eleven patients (61.1%) received END before SEMS placement. The median length of the stricture under fluoroscopy was 23 mm (range, 10–53 mm). The median procedure time was 25.0 min (range, 14–66 min). The diameters of SEMS used were 18 mm (22.2%) and 22 mm (77.8%). The lengths of SEMS used were 60 mm (11.1%), 80 mm (27.8%), 100 mm (27.8%), and 120 mm (33.3%), respectively. Seven patients (38.9%) received SEMS placement during their first procedure.

Technical and clinical success was achieved in all cases in the SEMS cohort. One patient with hepaticojejunal anastomosis stenosis required additional balloon dilation after four days. There were no early complications relating to SEMS placement. The median follow-up time was 102 days (range, 41–549 days). After SEMS placement, eleven patients (61.1%) received systemic chemotherapy. The median period of time to recurrent obstruction was not reached (95% CI: 119 days—not available (NA)). One late non-obstructive complication occurred in one patient who experienced abscess formation around the SEMS three month after SEMS placement. Two patients (11.1%) experienced ALO recurrence at 62 and 112 days after initial SEMS placement, respectively, both due to tumor or tissue ingrowth. Re-intervention with stent-in-stent placement of an additional partially covered SEMS was performed in one patient. The other patient was diagnosed with recurrent obstruction based on a dilated blind loop identified on abdominal ultrasound. Reflux cholangitis was treated conservatively with antibiotics, but the patient died due to progression of the primary disease one month later. There was no statistically significant difference in time to recurrent obstruction (*p* = 0.89) and overall survival (*p* = 0.06) with or without END placement before SEMS insertion. All patients died of their primary disease. The median overall survival was 102 days (95% CI: 62–180 days).

**Figure 3:**
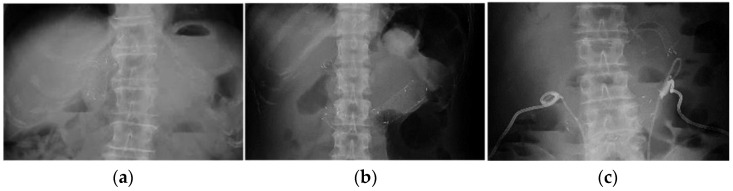
X-ray images of type 2 obstruction in the SEMS cohort (*n* = 3). (**a**) Endoscopic biliary self-expandable metal stent placement had already been performed before the placement of a self-expandable metal stent for malignant afferent loop obstruction. (**b**) Simultaneous endoscopic biliary and duodenal self-expandable metal stent placement. (**c**) Endoscopic ultrasound-guided hepaticogastrostomy was performed a few days after duodenal self-expandable metal stent placement.

Three cases were classified as type 2 obstruction in the SEMS cohort. In the first case, extensive left hepatectomy with Roux-en-Y reconstruction was performed for cholangiocarcinoma, and endoscopic biliary SEMS placement was performed using a BAE for malignant biliary obstruction following recurrence. Nine months later, malignant ALO occurred, and an uncovered SEMS was placed endoscopically. The second case was a patient with gastric cancer after total gastrectomy with Roux-en-Y reconstruction. Endoscopic transluminal SEMS placement was performed simultaneously for malignant biliary and duodenal obstruction using a BAE. The third case was a patient with gastric cancer with distal gastrectomy with Roux-en-Y reconstruction. Endoscopic transluminal SEMS placement was performed for malignant ALO, and EUS-guided hepaticogastrostomy (EUS-HGS) was performed for biliary drainage a few days later (Figure 3).

## 4. Discussion

In this study, we evaluated the results of endoscopic transluminal treatment for malignant ALO using a short-type BAE. The technical and clinical success rates of endoscopic transluminal treatment in the Initial cohort were both 95.5% (21/22), with only one early complication (4.5%) which was treated conservatively and evaluated as a mild adverse event. There were no early complications in the SEMS cohort. Recurrence of ALO occurred in two patients (11.1%) during a median follow-up period of 102 days, both due to tumor/tissue ingrowth. One was managed by additional SEMS placement and the other was treated conservatively. Abscess around the SEMS was observed in one patient as a late complication (5.6%), which was treated conservatively. No stent migration was occurred due to the use of uncovered SEMS. More than half patients (61.1%) received chemotherapy after SEMS placement without any stent-related complications.

Malignant ALO differs from other malignant gastrointestinal obstructions in several respects. Bile and pancreatic juices, but not solids such as food and feces, pass through the afferent loop. When the symptoms such as abdominal pain, fever, and jaundice appear, the blind loop is already markedly expanded and presents a risk of rupture, requiring urgent decompression. A decompression tube is often placed to avoid intestinal rupture, and END was selected as the initial treatment in 68.2% of patients in our study. Another difference is that there are various types of bowel reconstruction. Endoscopic transluminal treatment is particularly difficult when the reconstruction type is not known, requiring a consideration of other decompression methods such as PTCD.

Endoscopic transluminal SEMS insertion for malignant ALO with overtube-assisted insertion and TTS delivery has been reported in several studies (Table 4) [8,9,10,11,16,17,18,19,20,21,22,23,24,25,26,27,28,29,30,31,32]. Most previous reports are case reports, and reports on endoscopic SEMS placement in the setting of Roux-en-Y reconstruction are also limited. Information of long-term outcomes of endoscopic transluminal SEMS placement is therefore scarce. Technical and clinical success rates were reported to be 91–100%. The recurrent obstruction rate ranged between 0–38%. Kida et al. reported 11 patients with malignant ALO [11]. Among ten patients who achieved technical success, two patients (20.0%) had recurrent obstruction and received stent re-insertion 9 and 103 days after initial SEMS placement, respectively. Ito et al. reported ten patients with malignant ALO [31]. Two patients (20.0%) experienced recurrent obstruction, both of which were treated conservatively due to their poor general condition. In our study, two patients (11.1%) experienced recurrent obstruction in median follow-up period of 102 days. One patient was managed by additional SEMS placement and the other patient was treated conservatively due to poor general condition. Recently, Wang et al. reported a large multicenter retrospective study comparing covered and uncovered SEMS for malignant ALO [32]. The median time to stent dysfunction was 69 days, with no significant difference in stent dysfunction between the covered and uncovered SEMS groups. Stent ingrowth was more frequent in uncovered SEMS group, while stent migration was more frequent in covered SEMS group.

Recently, EUS-guided gastroenterostomy (EUS-GE) has been introduced as an alternative in the management of malignant ALO [33,34,35,36,37,38,39,40,41,42,43,44,45,46,47,48,49,50,51]. Technical and clinical success rates have been reported as 92–100% and 85–100%, respectively. There are two types of stents: lumen apposing metal stents (LAMS) and conventional SEMS. One-step insertion of LAMS is considered safer. EUS-GE is also associated with the risk of peritonitis due to leakage of bile and pancreatic juices. Bleeding risk should also be considered for patients taking antithrombotic agents. EUS-GE also proves difficult in patients who underwent gastrectomy or hepatectomy with Roux-en-Y reconstruction. Therefore, it is necessary to determine the optimal procedure on a case-by-case basis.

The management of type 2 obstruction is difficult in the cases of malignant ALO, especially when biliary obstruction occurs simultaneously or after enteral obstruction. Biliary cannulation may prove difficult because the papilla or bilioenteric anastomosis cannot be identified. EUS-HGS can be an effective biliary drainage method in such cases, although it may be necessary to resort to PTBD when EUS-HGS proves difficult. In our study, two patients had biliary SEMSs placed endoscopically, while one patient underwent EUS-HGS.

There are several limitations to this study. First, this was a single-center, single-arm, retrospective study with limited number of cases. Therefore, various biases may be involved. However, this study is second largest case series to date on this topic. Second, the sample population is rather heterogeneous, including various background factors such as primary disease, reconstruction type, ECOG PS, and stent types. Third, outcomes of covered SEMS placement and EUS-GS were not evaluated in this study.

## 5. Conclusions

High technical and clinical success was achieved by endoscopic transluminal treatment with short procedural times for malignant ALO. SEMS placement also appears to be safe and effective, and additional SEMS placement can be considered in cases of re-obstruction. Comparative studies with EUS-GE are required to identify the most desirable course of action in patients with malignant ALO.

## Figures and Tables

**Table 1:** Patient characteristics in Initial cohort (*n* = 22).

Age, Years, Median (Range)	64.5 (37–83)
Sex, *n* (%)	
Male	13 (59.1%)
Female	9 (40.9%)
Eastern Cooperative Oncology Group performance status, *n* (%)	
0	10 (45.5%)
1	8 (36.4%)
2	4 (18.2%)
Primary disease, *n* (%)	
Pancreatic cancer	10 (45.5%)
Biliary tract cancer	6 (27.3%)
Gastric cancer	5 (22.7%)
Esophageal cancer	1 (4.5%)
Type of surgery, *n* (%)	
Open	21 (95.5%)
Laparoscopic	1 (4.5%)
Type of reconstruction, *n* (%)	
Pancreaticoduodenectomy	11 (50.0%)
Gastrectomy with Roux-en-Y reconstruction	6 (27.3%)
Hepatectomy with Roux-en-Y reconstruction	3 (13.6%)
Double bypass	1 (4.5%)
Unknown (before the procedure)	1 (4.5%)
Ascites, *n* (%)	12 (54.5%)
Dilation of intrahepatic bile duct, *n* (%)	5 (22.7%)
Obstruction type, *n* (%)	
1	15 (68.2%)
2	4 (18.2%)
3	3 (13.6%)
Symptoms of ALO, *n* (%) *	
Abdominal pain	11 (50.0%)
Fever	9 (40.9%)
Elevated hepatobiliary enzymes or jaundice	4 (18.2%)
Nausea and vomiting	1 (4.5%)
Time from surgery to ALO, days, median (range)	612 (86–1806)
Use of antithrombotic agents, *n* (%)	5 (22.7%)
Prior treatment with anti-vascular endothelial growth factor antibody, *n* (%)	1 (4.5%)
Prior radiotherapy, *n* (%)	1 (4.5%)

ALO, afferent loop obstruction. * Total exceeds 100%, as multiple symptoms were observed in some patients.

**Table 2:** Endoscopic procedure outcomes of Initial cohort (*n* = 22).

Time to reaching the stenotic site, min, median (range)	10.5 (4–64)
Total procedure time, min, median (range)	28.0 (12–106)
Endoscopic passage through the stenotic site, *n* (%)	7 (31.8%)
Drainage method, *n* (%)	
END	15 (68.2%)
SEMS	7 (31.8%)
Technical success, *n* (%)	21 (95.5%)
Clinical success, *n* (%)	21 (95.5%)
Early complications, *n* (%)	1 (4.5%)
Micro-perforation	1 (4.5%)
Bleeding	0 (0.0%)
Stent migration	0 (0.0%)

END, endoscopic naso-drainage; SEMS, self-expandable metal stent.

**Table 3:** Endoscopic procedure and long-term outcomes of the SEMS cohort (*n* = 18).

END before SEMS placement, *n* (%)	11 (61.1%)
Length of the stricture, mm, median (range)	23 (10–53)
Total procedure time, min, median (range)	25.0 (14–66)
Technical success, *n* (%)	18 (100%)
Clinical success, *n* (%)	18 (100%)
Stent diameter, *n* (%)	
18 mm	4 (22.2%)
22 mm	14 (77.8%)
Stent length, *n* (%)	
60 mm	2 (11.1%)
80 mm	5 (27.8%)
100 mm	5 (27.8%)
120 mm	6 (33.3%)
Early complications, *n* (%)	0 (0.0%)
Follow-up period, days, median (range)	102 (41–549)
Late complications, *n* (%)	
Abscess around the SEMS	1 (5.6%)
Stent migration	0 (0.0%)
Recurrence of ALO, *n* (%)	2 (11.1%)
Re-intervention, *n* (%)	1 (5.6%)
Received chemotherapy after SEMS placement, *n* (%)	11 (61.1%)
Time to recurrent obstruction, days, median (95% CI)	NA (119–NA)
Overall survival, days, median (95% CI)	102 (62–180)

END, endoscopic naso-drainage; SEMS, self-expandable metal stent; ALO, afferent loop obstruction, 95% CI, 95% confidence interval; NA, not available.

**Table 4:** Previous reports of endoscopic transluminal self-expandable metal stent placement for malignant afferent loop obstruction.

Authors	Year	*n*	Type of Reconstruction	Type of SEMS	TSR (%)	CSR (%)	EC (%)	TTS/OA, (*n*)	GIE/BAE (*n*)	Recurrent Obstruction Rate, (%)
			B-II/PD/RY/Other (*n*)	UC/C (*n*)						
Burdick et al.	2002	1	1/0/0/0	NA	100	100	0	NA	NA	NA
Kim et al.	2011	2	0/2/0/0	2/0	100	100	0	2/0	2/0	NA
Kida et al.	2013	1	0/1/0/0	NA	100	100	0	0/1	0/1	NA
Sasaki et al.	2014	1	0/1/0/0	1/0	100	100	0	0/1	0/1	NA
Kwong et al.	2014	2	0/2/0/0	2/0	100	100	0	NA	NA	0
Shugo et al.	2015	1	0/1/0/0	1/0	100	100	0	0/1	1/0	0
Sakai et al.	2015	1	0/1/0/0	1/0	100	100	0	1/0	1/0	0
Fujii et al.	2015	2	0/1/1/0	2/0	100	100	0	0/2	0/2	0
Huang et al.	2015	3	0/3/0/0	3/0	100	100	0	3/0	3/0	0
Nakahara et al.	2015	3	0/2/1/0	3/0	100	100	0	0/3	0/3	0
Nakahara et al.	2016	1	1/0/0/0	1/0	100	100	0	1/0	NA	0
Shimataniet al.	2016	1	0/1/0/0	1/0	100	100	0	1/0	0/1	NA
Minaga et al.	2016	1	0/1/0/0	1/0	100	100	0	1/0	0/1	NA
Kannno et al.	2018	4	0/3/1/0	4/0	100	100	0	4/0	2/2	0
Takeuchi et al.	2018	1	0/0/1/0	1/0	100	100	0	1/0	1/0	0
Sasaki et al.	2018	5	0/4/1/0	5/0	100	100	0	4/0	0/5	NA
Yane et al.	2018	5	0/4/1/0	5/0	100	100	0	1/4	0/5	0
Sakai et al.	2020	7	0/7/0/0	7/0	100	100	0	7/0	7/0	0
Kida et al.	2020	11 *	3/7/1/0	10/0	91	91	0	9/1	6/5	20.0%
Ito et al.	2022	10	0/NA/NA/0	10/0	100	100	0	10/0	0/10	20.0%
Wang et al.	2022	137	NA/76/NA/0	72/65	100	95	10.9%	137/0	137/0	38.0%
Our study		18	0/10/7/1 **	18/0	100	100	0	18/0	0/18	11.1%

B-II, Billroth-II reconstruction; PD, pancreaticoduodenectomy; R-Y, Roux-en-Y reconstruction; SEMS, self-expandable metal stent; UC, uncovered; C, covered; TSR, technical success rate; CSR; clinical success rate; EC; early complication; TTS, through-the-scope; OA, overtube-assisted; GIE, gastrointestinal endoscope; BAE, balloon-assisted enteroscope; NA, not available. * Includes one technical failure case; ** Double bypass.

## Data Availability

The data that support the finding of this study are available from the corresponding author, T.S., upon reasonable request.
